# Extensive prediction of drug response in mutation-subtype-specific LUAD with machine learning approach

**DOI:** 10.32604/or.2023.042863

**Published:** 2023-12-28

**Authors:** KEGANG JIA, YAWEI WANG, QI CAO, YOUYU WANG

**Affiliations:** 1 Department of Thoracic Surgery, Sichuan Provincial People’s Hospital, University of Electronic Science and Technology of China, Chengdu, China; 2 School of Medicine, University of Electronic Science and Technology of China, Chengdu, China; 3 Department of Assisted Reproductive Medicine, Sichuan Provincial Academy of Medical Sciences & Sichuan Provincial People’s Hospital, Chengdu, China

**Keywords:** Lung adenocarcinoma, Drug resistance, Machine learning, Molecular features, Personalized treatment

## Abstract

**Background:**

Lung cancer is the most prevalent cancer diagnosis and the leading cause of cancer death worldwide. Therapeutic failure in lung cancer (LUAD) is heavily influenced by drug resistance. This challenge stems from the diverse cell populations within the tumor, each having unique genetic, epigenetic, and phenotypic profiles. Such variations lead to varied therapeutic responses, thereby contributing to tumor relapse and disease progression.

**Methods:**

The Genomics of Drug Sensitivity in Cancer (GDSC) database was used in this investigation to obtain the mRNA expression dataset, genomic mutation profile, and drug sensitivity information of NSCLS. Machine Learning (ML) methods, including Random Forest (RF), Artificial Neurol Network (ANN), and Support Vector Machine (SVM), were used to predict the response status of each compound based on the mRNA and mutation characteristics determined using statistical methods. The most suitable method for each drug was proposed by comparing the prediction accuracy of different ML methods, and the selected mRNA and mutation characteristics were identified as molecular features for the drug-responsive cancer subtype. Finally, the prognostic influence of molecular features on the mutational subtype of LUAD in publicly available datasets.

**Results:**

Our analyses yielded 1,564 gene features and 45 mutational features for 46 drugs. Applying the ML approach to predict the drug response for each medication revealed an upstanding performance for SVM in predicting Afuresertib drug response (area under the curve [AUC] 0.875) using CIT, GAS2L3, STAG3L3, ATP2B4-mut, and IL15RA-mut as molecular features. Furthermore, the ANN algorithm using 9 mRNA characteristics demonstrated the highest prediction performance (AUC 0.780) in Gefitinib with CCL23-mut.

**Conclusion:**

This work extensively investigated the mRNA and mutation signatures associated with drug response in LUAD using a machine-learning approach and proposed a priority algorithm to predict drug response for different drugs.

## Introduction

Lung cancer is the leading cause of cancer-related deaths and one of the most commonly diagnosed malignancies globally [1]. Approximately 85% of lung cancer patients are diagnosed with Non-Small Cell Lung Cancer (NSCLC). Within this category, the dominant histological subtypes are lung adenocarcinoma (LUAD) and lung squamous cell carcinoma (LUSC) [2,3]. Treatment options for non-small cell lung cancer include surgical resection, chemotherapy, radiation, targeted therapy, immune therapy, and or a combination of these. Polychemotherapy for NSCLC frequently consists of a platinum-based drug (such as cisplatin or carboplatin) combined with additional treatments with a different action mechanism [3]. Chemotherapy and radiation treatment have been shown to extend the time to progression and Overall Survival (OS) in patients with inoperable stage III NSCLC [4]. However, drug resistance is a primary culprit for therapeutic failure in NSCLC, culminating in tumor recurrence and disease progression. This resistance stems from the tumor’s diverse cell population, each possessing varied genetic, epigenetic, and phenotypic traits, leading to a spectrum of responses to treatment.

Research evidence indicates that the LUAD subtype be considered when selecting a chemotherapeutic drug [5–7]. Veronesi et al. [5] revealed that second-line paclitaxel therapy was more effective in LUAD following cisplatin administration for lung cancer. Gemcitabine-platinum and taxane-platinum regimens tend to increase median survival time for adenocarcinoma, albeit the former has higher objective response rates and a trend to improve OS [7]. High-throughput sequencing technology and bioinformatics approaches have been applied to elucidate disease pathogenesis [8,9] and highlight the underlying genetic features that define subtypes of lung cancer [10,11]. These cutting-edge technologies aided in the discovery of a complex network of driver mutations that are intricately linked to varied therapy response rates. This correlation, underscored by the multifaceted interplay between genetic abnormalities and therapeutic efficacy, provides vital insights for tailoring precision medicine strategies, enhancing therapeutic outcomes, and expanding our general understanding of lung cancer biology. EGFR TKIs have a high response rate (60%–70%) in EGFR-mutated cancers [12], while Anaplastic Lymphoma Kinase (ALK) inhibitors have a comparable response rate (60%) in patients with ALK translocations [13].

The significance of immunological research in cancer has grown in recent years. A deeper understanding of immune responses, particularly in the context of LUAD, might provide valuable insights into disease pathogenesis and therapeutic intervention. Like many cancers, LUAD manipulates the immune system to promote tumor progression and resistance to therapy. Research evidence has demonstrated a complex interplay between tumor cells, immune cells, and the tumor microenvironment in LUAD, leading to immune evasion and disease progression. The role of sepsis-associated genes in LUAD has particularly piqued interest from researchers due to their potential as therapeutic targets. Sepsis-associated genes play vital roles in immunological responses, and their dysregulation can result in immune dysfunction and contribute to LUAD pathogenesis. Furthermore, immune-based therapeutic strategies, including immunotherapy, have shown promise in LUAD treatment, emphasizing the relevance of immunological research. Understanding the complex interactions between immune response, sepsis-associated genes, and LUAD may give new insights into disease mechanisms and pave the way for developing innovative, targeted therapies.

Additionally, Machine Learning (ML), a specific subset of artificial intelligence that enables autonomous learning from data, has significantly contributed to genomics research [14–16]. Several studies have used ML-based approaches to predict treatment responses in various diseases [17,18]. For instance, Ahn et al. (2021) used ML to develop a clinical decision support algorithm to predict the anti-PD-1 response in LUAD [18]. However, most research focuses on predicting the response to a specific treatment and providing an appropriate strategy. Extensive drug response prediction and prediction accuracy comparison between ML-based methods are warranted because various treatments adapt to different approaches to estimate the response rate.

**Figure 1:**
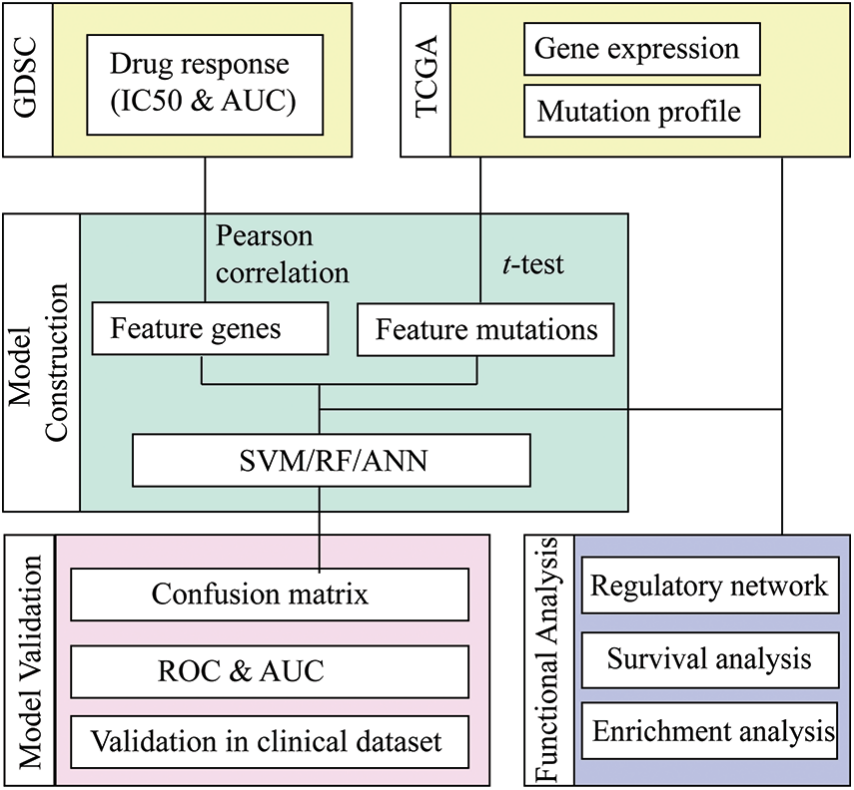
Workflow of drug response prediction in mutation-subtype-specific LUAD with machine learning approach.

The present investigation employed the genomics of drug sensitivity in cancer (GDSC) database to retrieve the mRNA expression dataset, genomic mutation profile, and drug sensitivity information of NSCLS. Machine learning methods, including Random Forest (RF), Artificial Neurol Network (ANN), and Support Vector Machine (SVM), were applied for each compound to predict the response status based on the mRNA and mutation features selected by statistical methods. The most suitable method for each drug was proposed by comparing the prediction accuracy of different ML methods, and the selected mRNA and mutation features were identified as molecular features for the drug-responsive cancer subtype (Fig. 1).

## Materials and Methods

### Data resource

The mRNA expression datasets, gene mutation profile, and drug response sensitivity of LUAD were downloaded from Genomics of Drug Sensitivity in Cancer (GDSC) [19]. A total of 55 cell lines with the treatment of 175 drug compounds were analyzed, and 50% inhibitory concentration (IC50) and Area Under Curve (AUC) were retrieved to represent the drug sensitivity of cell lines. In addition, 37,263 genes with Transcripts Per Kilobase Million (TPM) value were extracted, and a mutation profile containing 19,913 genes was included.

### Identification of drug response-related genes

The Pearson correlation coefficient was calculated between the values of IC50/AUC and the expression of each gene for each drug to select gene features for response prediction. A gene correlated to IC50 with |cor| > 0.5 and correlated to AUC with |cor| > 0.5 was deemed a drug response-related gene.

### Identification of drug response-related mutation

To select feature mutations for response prediction, the corresponding cell lines for each drug were divided into mut-type groups and wild-type groups based on the mutation status of the gene. *t*-test was then applied to determine the significance of differences between mut-type and wild-type groups. *p*-values were determined, and *p*-value < 0.05 were used to screen out significantly different pairs. *p*-values of *t*-test of IC50 < 0.05 and AUC < 0.05 denoted drug response related mutations.

### Survival analysis

We retrieved the mRNA profiles and clinical data of LUAD samples from the TCGA database. The survival risk of patients was predicted using a Cox proportional hazards model-based survival analysis. Patients were divided in half based on the median gene expression levels of a selected marker gene. The R program “Survival” [20] was used to perform a Kaplan-Meier curve analysis, and the *p*-value between the two groups was determined. Marker genes were described as significantly correlated indicators for LUAD prognosis (*p* < 0.05).

### Pathway enrichment analysis

The Gene Set Enrichment Analyze (GSEA) [21] of biological processes was performed using the Gene Set Variation Analysis (GSVA) [22] R package. GO enrichment analysis [23] and KEGG enrichment analysis [24] were performed using the clusterProfiler [25] R package. The scores of immune-related pathways were quantified using the GSEA technique to investigate immune-relevant biological processes; The genes implicated in these pathways were retrieved from the Molecular Signatures Database (MSigDB) [26].

### Prediction of drug response with machine learning algorithm

Three ML approaches (including RF, SVM, and ANN) were employed to predict drug response using R software. The dataset was divided into a training dataset (70%) and a test dataset (30%) for each compound. Subsequently, experimentally measured IC50 values (the half-maximal inhibitory concentration of a compound with respect to cell viability) were used to train RF/SVM/ANN classification models that predict IC50 of LUAD cell lines against each compound, allowing for drug response prediction. The prediction model was trained using a subset of the full data matrix containing gene and mutation features. Finally, 10-fold cross-validation was applied, and the confusion matrix and AUC of the prediction model for train and test data were calculated to measure the prediction efficiency.

### Detection of cell apoptosis levels by flow cytometry

Cells are inoculated into a 6-well plate and washed twice with PBS, then resuspended in 500 μL of sample buffer. According to the instructions of the kit, the cells are labeled with Annexin V-FITC/PI and then detected by flow cytometry.

### Cell cycle detection method

Cells (A549 and NCI-H1975) in the logarithmic growth phase are used, adjusting the cell density to 1 × 105/mL. The cells are inoculated into a 6-well plate at 2 × 105 cells/well. siRNAs transfection and hypoxia treatments are conducted as previously described. Each group of cells is digested with trypsin, collected, and washed once with PBS. They are then added to 1 mL of DNA staining solution containing 10 μL of permeabilizing solution, and the mixture is vortexed for 5–10 s for thorough mixing. After incubation at room temperature, in the dark, for 30 min, the cell cycle is detected by flow cytometry (n = 3 per group).

### Hoechst 33342 staining of live cells

Cells are inoculated into a 6-well plate. After the cells are cultured post si-1 and si-2 intervention, the culture medium is discarded. The cells are fixed with ethanol, washed twice with PBS, and stained with Hoechst 33342 stain for 15 min. After washing twice with PBS, the cells are mounted with glycerol water solution, and observed under a fluorescence microscope. The cell nuclei appear as blue fluorescence.

### Detection of protein expression levels by western blot

Cells are inoculated into a 6-well plate and cultured post si-1 and si-2 intervention. After the culture medium is discarded and cells are collected, the cells are lysed on ice for 30 min with lysis buffer. After the lysis solution is clarified by centrifugation, the supernatant is collected, and protein quantification is performed using a BCA kit. The protein concentration is adjusted, followed by SDS-PAGE and PVDF transfer. The membrane is blocked, and primary antibodies (p-AKT and p-PI3K, diluted 1:500 and 1:800 respectively) are added, and the solution is incubated overnight at 4°C. After washing twice with TBST, secondary antibodies are added (diluted 1:3000), and the reaction is developed using an ECL kit. The grayscale value is analyzed using Image J software.

### Detection of mRNA expression levels by RT-qPCR

Cells are inoculated into a 6-well plate and cultured post si-1 and si-2 intervention for 24 h. After the culture medium is discarded and cells are collected, total RNA is extracted using the Trizol method. The reaction system is prepared according to the instructions of the kit, and the reaction is performed at 37°C for 60 min, reverse transcribing mRNA into cDNA. The PCR reaction system is prepared according to the real-time fluorescence quantitative PCR kit: 2.5 × RealMaster Mix/20 × SYBR solution 4.5 μL, 1 μL each of the forward and reverse primers, cDNA 2 μL, and nuclease-free water 1.5 μL, total 10 μL. The primer sequences are given in Table 1. The reaction conditions are as follows: initial denaturation at 94°C for 15 min, followed by 40 cycles of denaturation at 94°C for 20 s, annealing at 56°C for 30 s, and extension at 68°C for 30 s. GAPDH is used as an internal control to calculate the expression of the target RNA.

**Table 1:** Primer of KREMEN2 used in this study

Gene	Forward sequence (5′ to 3′)	Reverse sequence (5′ to 3′)
KREMEN2	CACAACTTCTGCCGTAACCC	CACAAAGCATCCCAGGTAGC

### Statistical analysis

Unless otherwise stated, all analyses and visualisations were performed using R software (version 4.2.1). Statistical significance was set at *p* < 0.05.

### Gene features for drug-response prediction

**Figure 2:**
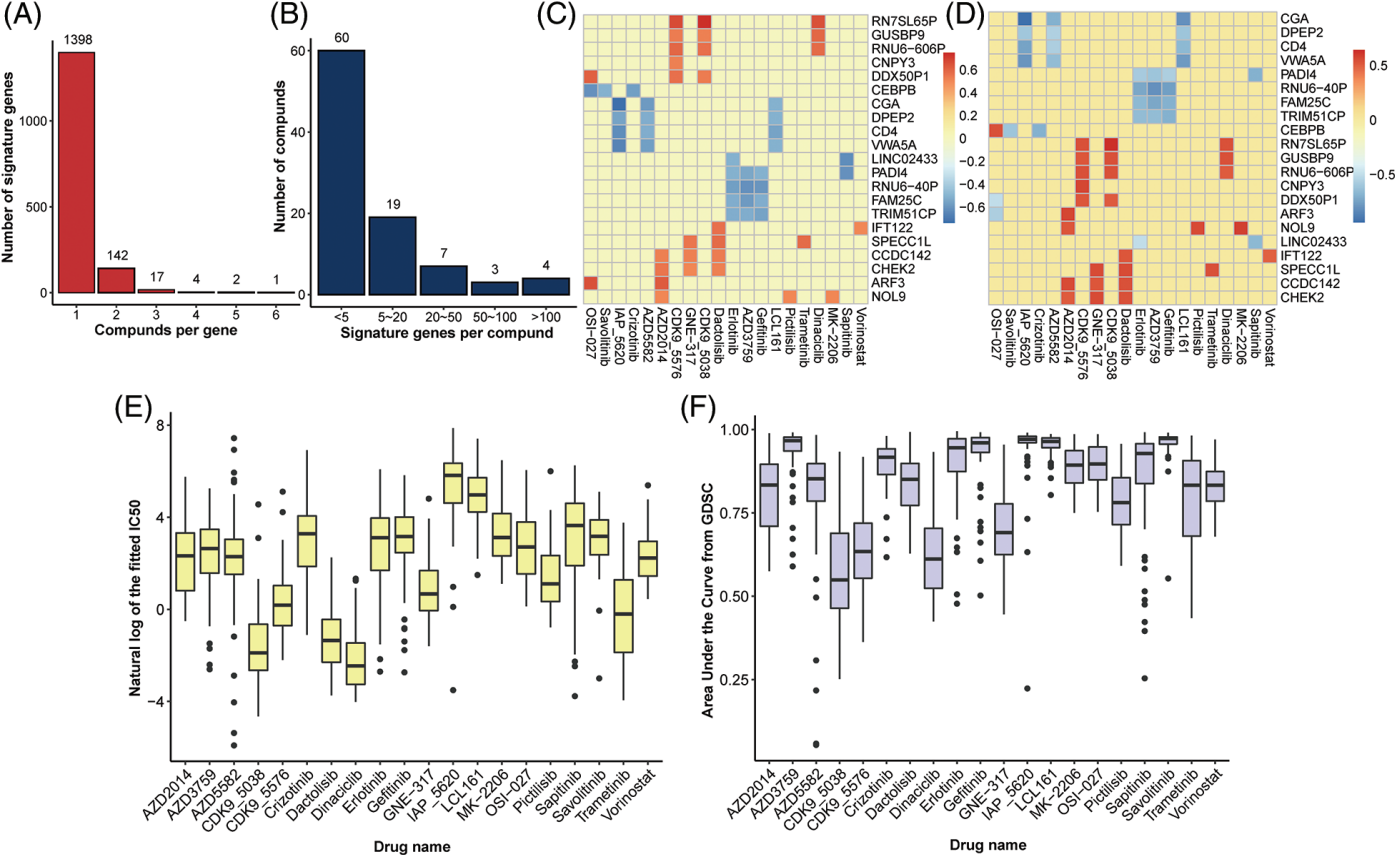
Identification of gene signatures. Distribution of (A) drugs per signature gene and (B) signature genes per drug. Heatplot of the correlation coefficient between expression of signature genes and (C) IC50 and (D) AUC form GDSC (signature genes per drug >5, drugs per signature gene >3), respectively. (E) Boxplot of nature log of the fixed IC50 and (F) AUC in drugs with at least one signature gene.

A total of 175 drug compounds corresponding to 55 lung cancer cell lines from GDSC were analyzed, and gene features for each drug were identified according to Method. As a result, 47.86% (82/175) of drugs lacked gene features and were thus excluded in downstream analysis. Most drugs (26.88%, 25/93) contained one gene feature, whereas 90.52% (86/95) of the drugs had <10 characteristic genes (Fig. 2A). OSI-027 is an orally bioavailable mammalian target of rapamycin (mTOR) kinase inhibitor with potential antineoplastic activity [27] and has the most characteristic genes of any medication (416). Besides, Savolitinib has 263 characteristic genes and has been shown to promote anti-tumour activity in patients with MET-amplified, EGFR mutation-positive, advanced LUAD [28]. On the other hand, most genes (89.39%, 1398/1564) are solely characteristics of one drug, implying various response mechanisms for different drugs (Fig. 2B).

### Mutation features for drug response prediction

**Figure 3:**
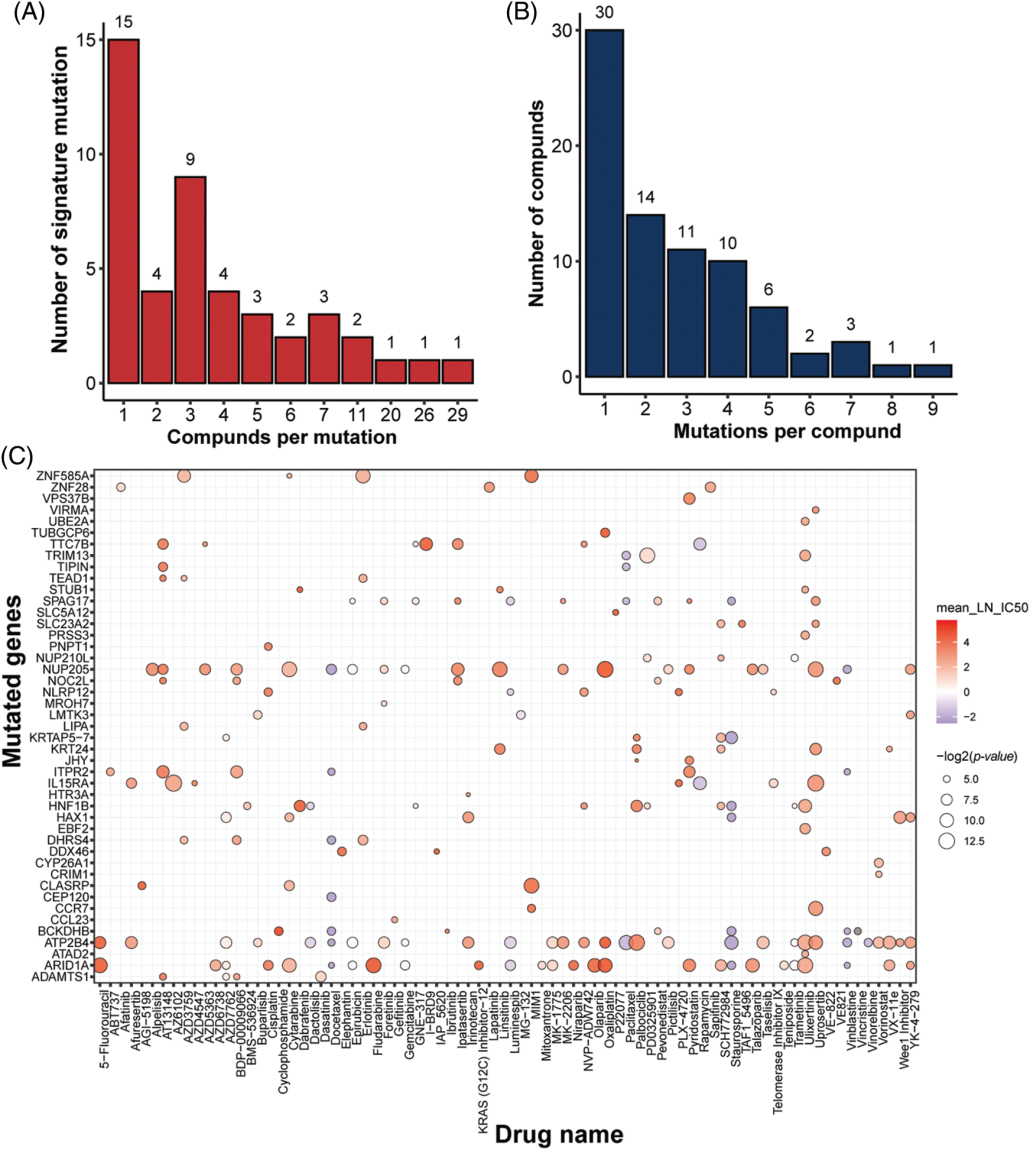
Identification of mutation signatures. Distribution of (A) drugs per signature mutation and (B) signature mutations per drug. (C) overview of all the signature mutations for all drugs with *p*-value (IC50) < 0.05 and *p*-value (AUC) < 0.05.

**Figure 4:**
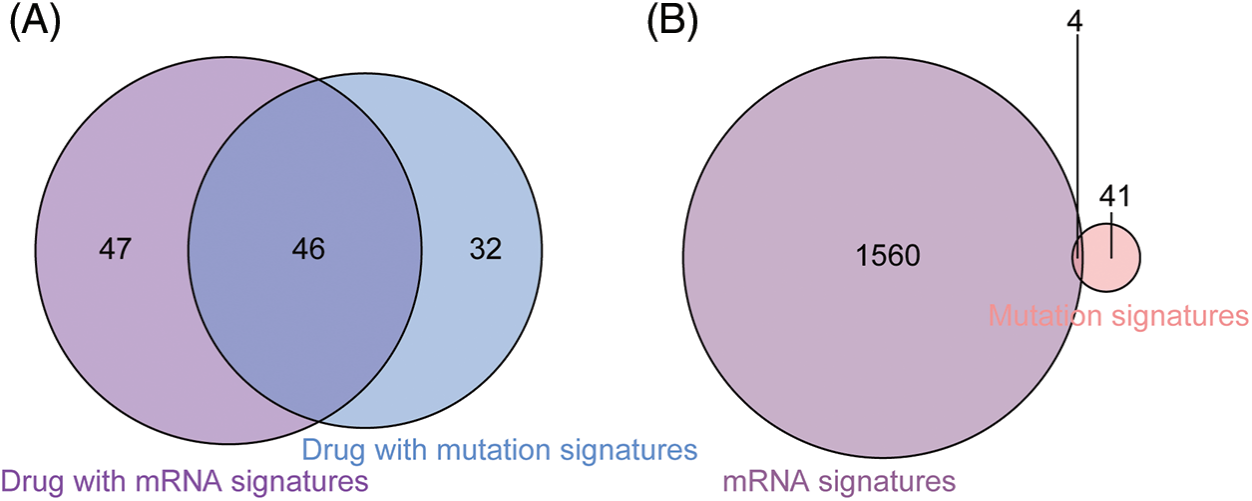
Comparison between drugs with gene signatures and mutation signatures. (A) Forty-six drugs included both signature gens and signature mutations. (B) The mutatation status of 4 signature genes was identified as signature mutations.

In addition to gene features, mutation characteristics played a role in model construction, and 45 mutant genes were identified as characteristic mutations for 78 drugs. A single characteristic mutation existed in 38.46% (30/78) of all the drugs (Fig. 3A). Ulixertinib and Uprosertib had the most and second-most characteristic mutations among all the drugs, with 9 and 8, respectively. Ulixertinib (BVD-523) is an ERK1/2 kinase inhibitor with potent preclinical activity in BRAF- and RAS-mutant cell lines [29]. Furthermore, 22.22% (10/45) of the mutations were identified as characteristic mutations for at least 5 drugs, whereas 33.33% (15/45) of the mutations were identified as characteristic mutations in one drug (Fig. 3B). The mutant status of ATP2B4 was significantly correlated with the drug response of 29 compounds (Fig. 3C). Previous research revealed that p38 MAPK activation induces internalization and subsequent degradation of ATP2B4 through the endo/lysosomal system, contributing to the low ATP2B4 steady-state protein level and making it a putative metastasis suppressor in BRAF mutant melanoma [30]. Ninety-three compounds with 1564 feature genes and 78 compounds with 45 feature mutations were selected for downstream analysis using the feature selection method. Furthermore, 46 compounds had gene and mutation features used to construct the prediction model (Fig. 4).

### Drug response prediction

**Figure 5:**
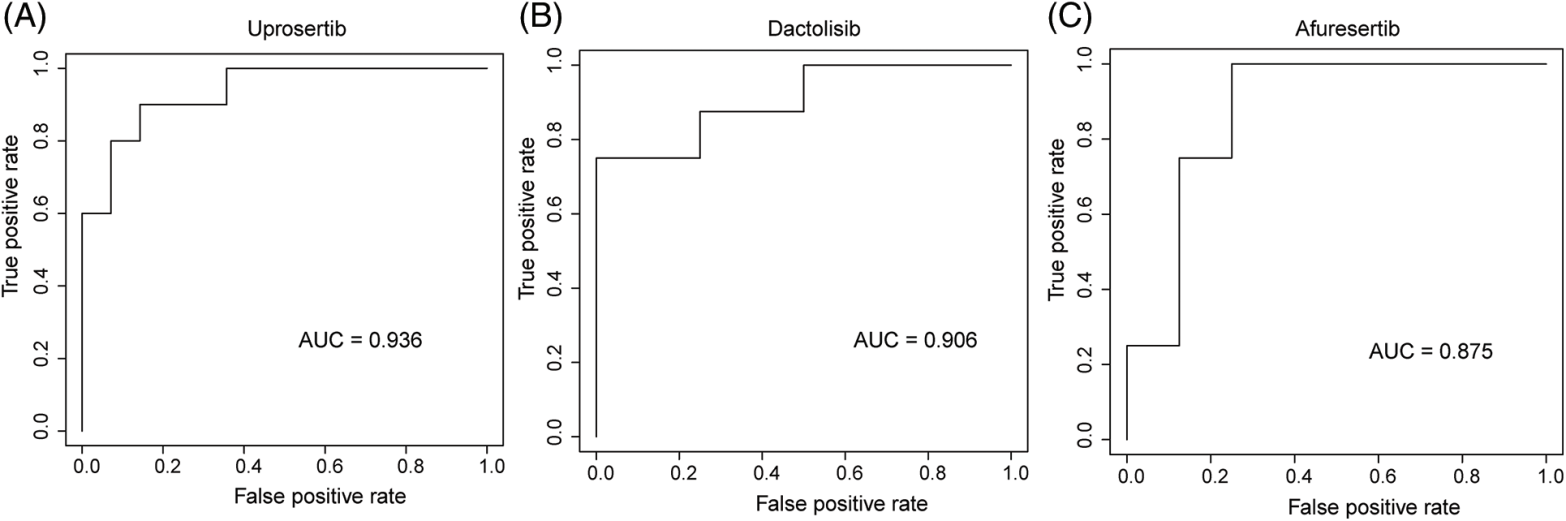
AUC of (A) Upeosertib, (B) Dactolisib, (C) Afuresertib model with SVM algorithm.

**Figure 6:**
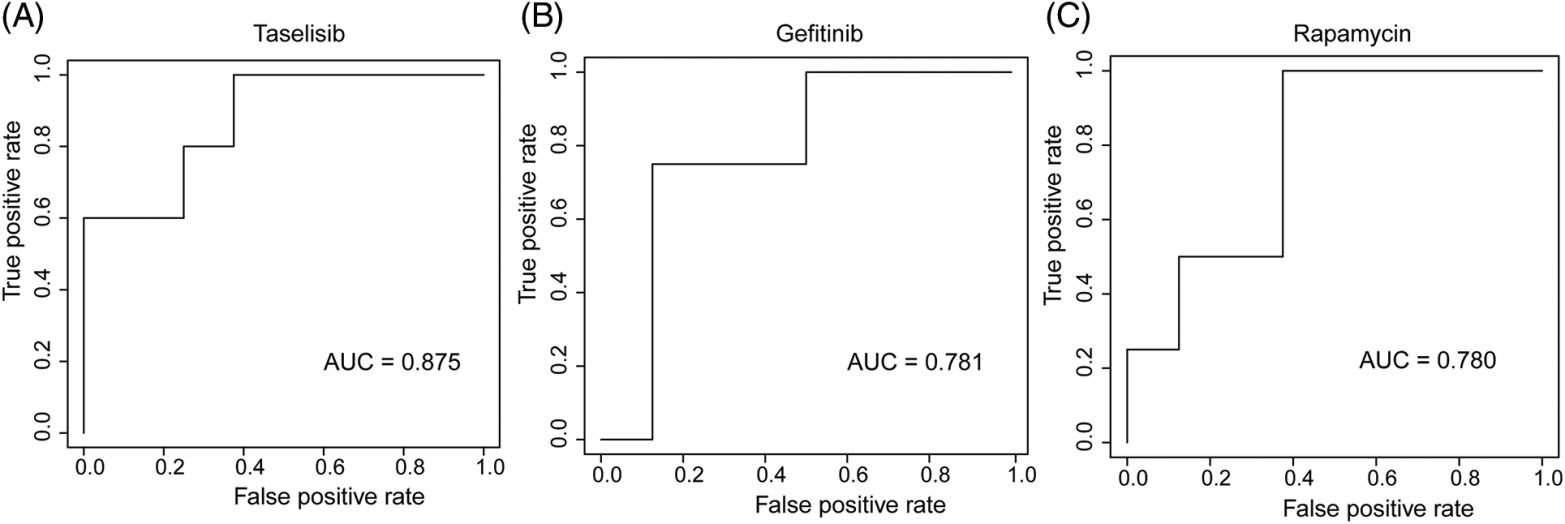
AUC plot of (A) Taselisib, (B) Geftinib, and (C) Rapamycin model with ANN algorithm.

**Figure 7:**
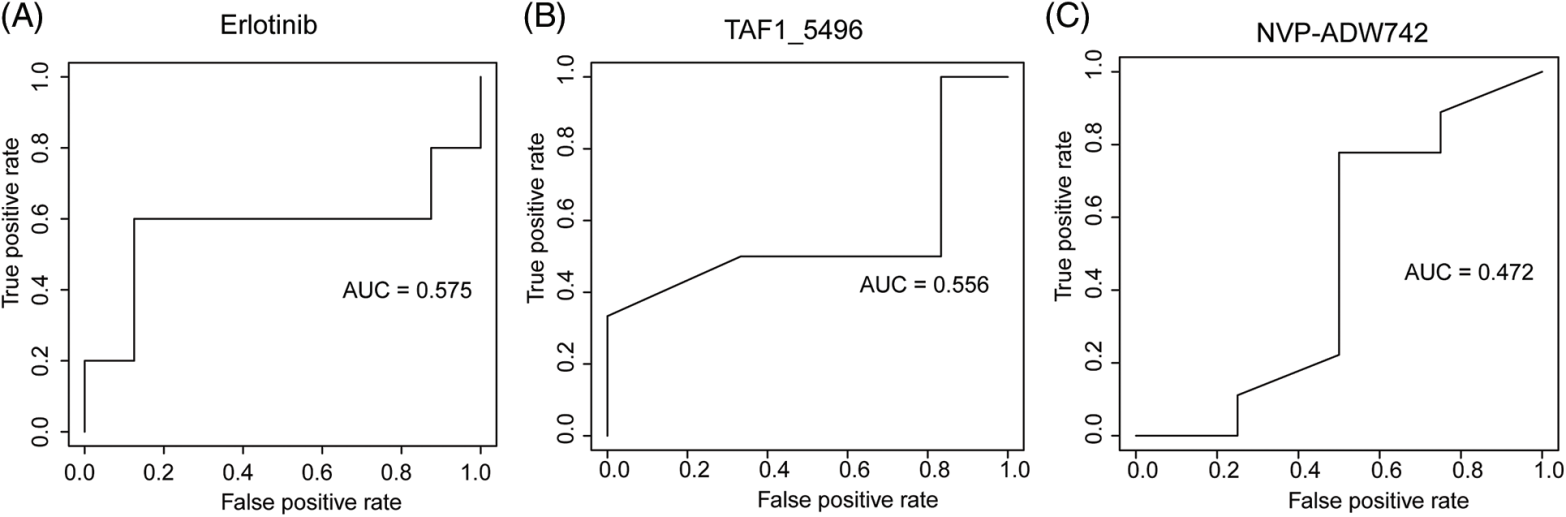
AUC plot of (A) Erlotinib, (B) TAF1_5496, (C) NVP-ADW742 model with RF algorithm.

We employed three machine learning algorithms to develop prediction models for 46 drugs containing both drug and mutation characteristics to predict the response of LUAD to drugs based on the screened genes and mutation characteristics related to drug response. The AUC value of each model was determined to assess the prediction capacity of different algorithms for different drug responses. SVM (Support Vector Machine) is a widely used supervised binary classification model, and it showed upstanding performance in the response prediction model for 11 drugs. Uprosertib (AUC = 0.94) (Fig. 5A) demonstrated stronger predictive power than other drugs, followed by Dactolisib (AUC = 0.90) (Fig. 5B), Afuresertib (AUC = 0.88) (Fig. 5C). Furthermore, we used the neuralnet package of R to construct an ANN model for drug response prediction and calculated the accuracy and AUC value of each model. The ANN model with accuracy, specificity, and AUC value > 0.5 is used as an effective model, and 17 effective ANN models are obtained: Telomerase Inhibitor IX (AUC = 0.97), BMS-536924 (AUC = 0.91), Taselisib (AUC = 0.87) (Fig. 6A), Vinorelbine (AUC = 0.87), TAF1_5496 (AUC = 0.85), Gefitinib (AUC = 0.78) (Fig. 6B), Rapamycin (AUC = 0.78) (Fig. 6C), Dactolisib (AUC = 0.75), Erlotinib (AUC = 0.74), Vorinostat (AUC = 0.74), Mitoxantrone (AUC = 0.70), VX-11e (AUC = 0.65), Trametinib (AUC = 0.61), BDP-00009066 (AUC = 0.59), YK-4-279 (AUC = 0.58), SCH772984 (AUC = 0.57), Vinblastine (AUC = 0.54). The RF algorithm is a decision tree-based classifier ensemble algorithm. We constructed a response RF model for each drug using the randomForest package in R. The model verification findings revealed that the RF algorithm has a poor prediction effect, with the Erlotinib response model having the best AUC value of 0.58 (Fig. 7). To summarize, a comparison of the drug response models of the three machine learning algorithms demonstrated SVM as the best algorithm for constructing a drug response model for LUAD, with an average value of 0.74 for the 46 drug response prediction models, followed by ANN (with an average AUC value of 0.66), and the RF algorithm performed the worst, with an average AUC of 0.23.

### Prognosis analysis for signature genes and mutations

**Figure 8:**
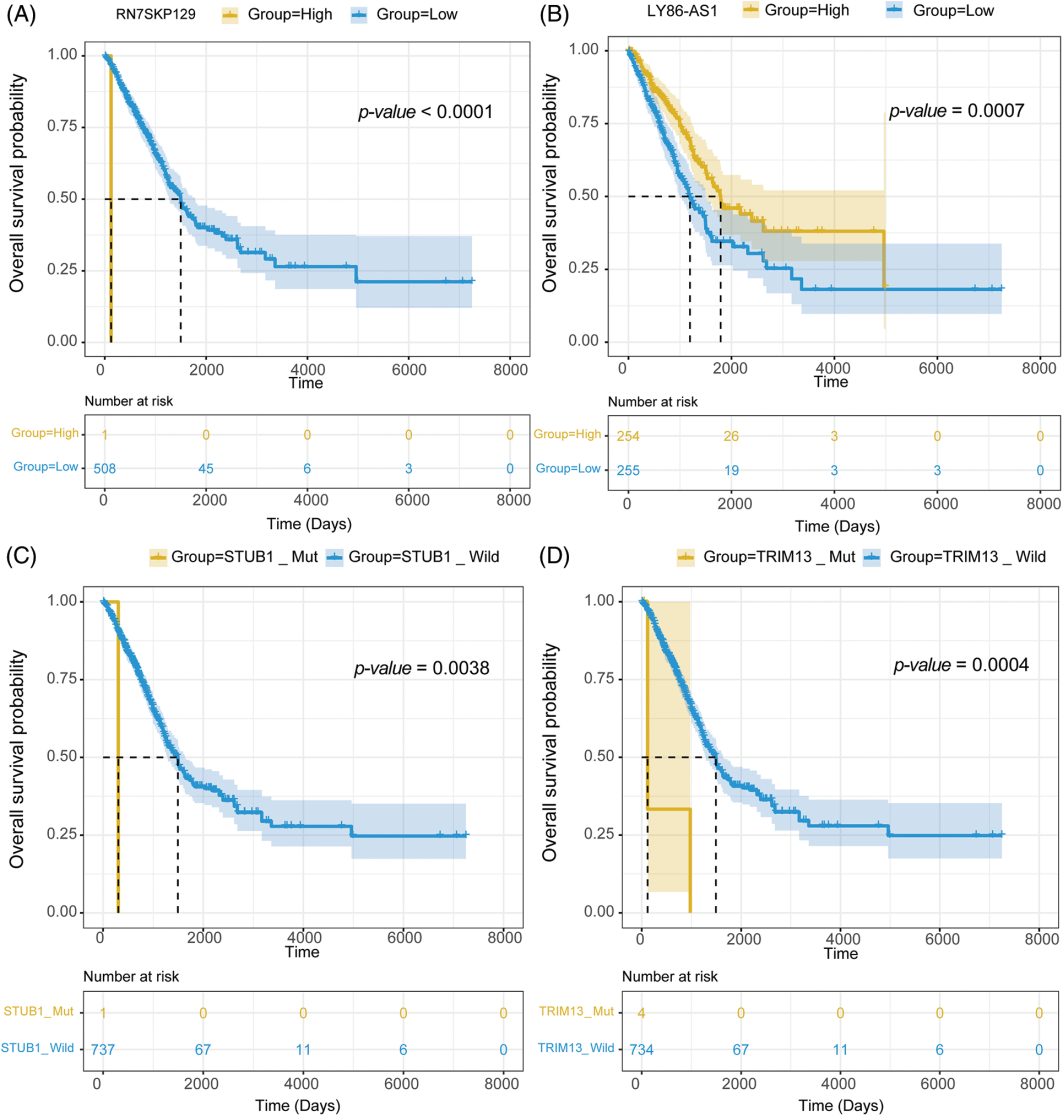
Survival analysis of (A) RN7SKP129, (B) LY86-AS1, (C) mut-STUB, and (D) mut-TRIM13 in LUAD with TCGA dataset.

mRNA expression matrix and clinical information from TCGA-LUAD were collected. Survival analysis was then conducted on 1,564 signature genes and 45 mutations to further examine the impact of drug response predictive gene and mutation signatures on LUAD prognosis. Survival analysis revealed that 111 signature genes for 36 drugs were significantly linked to LUAD prognosis. RN7SKP129, the signature gene of Savolitinib, was the most significant gene related to LUAD prognosis (*p*-value = 0.00000053) (Fig. 8A), and patients with high expression of RN7SKP129 had a better prognosis. Besides, LY86-AS1 was one of the characteristic genes of IAP-5620, and survival analysis revealed a significantly worse prognosis of LUAD patients with high expression of LY86-AS1 (*p*-value = 0.00070) (Fig. 8B). The rest of the top 10 significant prognosis-related signature genes included MED15P9 (*p*-value = 0.00099) for OSI-027, MYO1H (*p*-value = 0.0013) for AZD2014, MYEOV (*p*-value = 0.0014) for OSI-027, CD83P1 (*p*-value = 0.0021) for AZD5991 and TAF1_5496, TFAP2A (*p*-value = 0.0026) for IAP-5620, KREMEN2 (*p*-value = 0.0029), TRAV34 (*p*-value = 0.0032) for Savolitinib, and PIMREG (*p*-value = 0.0033) for I-BET-762. mut-STUB1 (*p*-value = 0.0038) (Fig. 8C), TRIM13 (*p*-value = 0.0002) (Fig. 8D), mut-HTR3A (*p*-value = 0.039), mut-BCKDHB (*p*-value = 0.039), TUBGCP6 (*p*-value = 0.002), and mut-LIPA (*p*-value = 0.00015) were identified as feature mutations and significantly related to LUAD prognosis.

### Pathways related to drug-response activities

**Figure 9:**
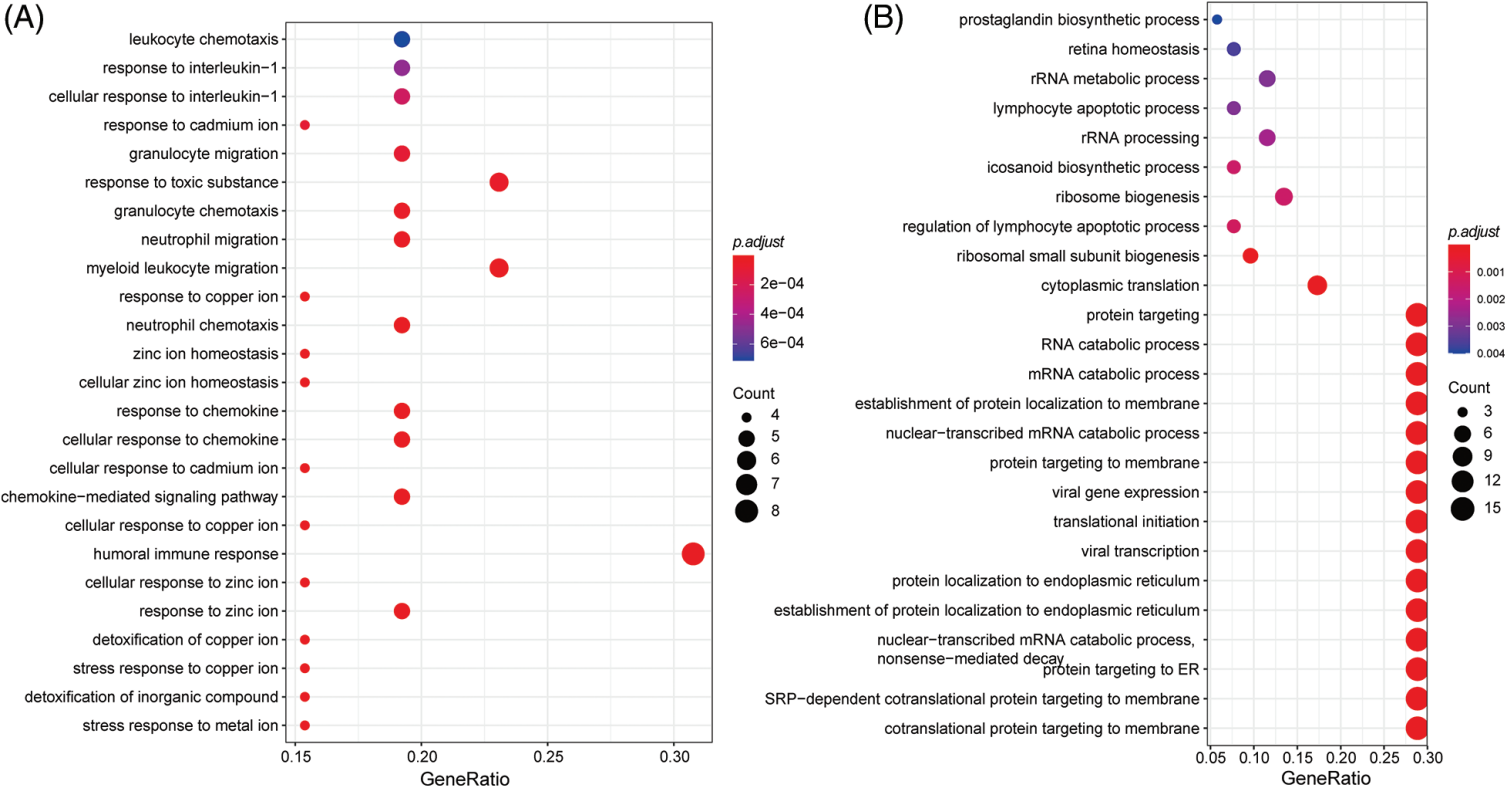
GO enrichment analysis of signatures genes.

Genes with specific expression play a pivotal role in drug response dynamics. To delve into the molecular mechanisms affecting LUAD drug response, we conducted an enrichment analysis on these signature genes. The GO enrichment analysis showed that the signature genes predominantly participated in immune-related pathways, such as the “cellular response to chemokine” (Fig. 9A). They were also notably involved in ion transportation pathways, highlighting the “stress response to metal ion,” “response to zinc ion,” and “cellular response to copper ion” (Fig. 9B). The enrichment outcomes align well with our understanding of cell signaling within the LUAD microenvironment, suggesting intricate functional mechanisms governing the LUAD response to drug treatment”.

### The impact of siRNA on LUAD cell apoptosis

**Figure 10:**
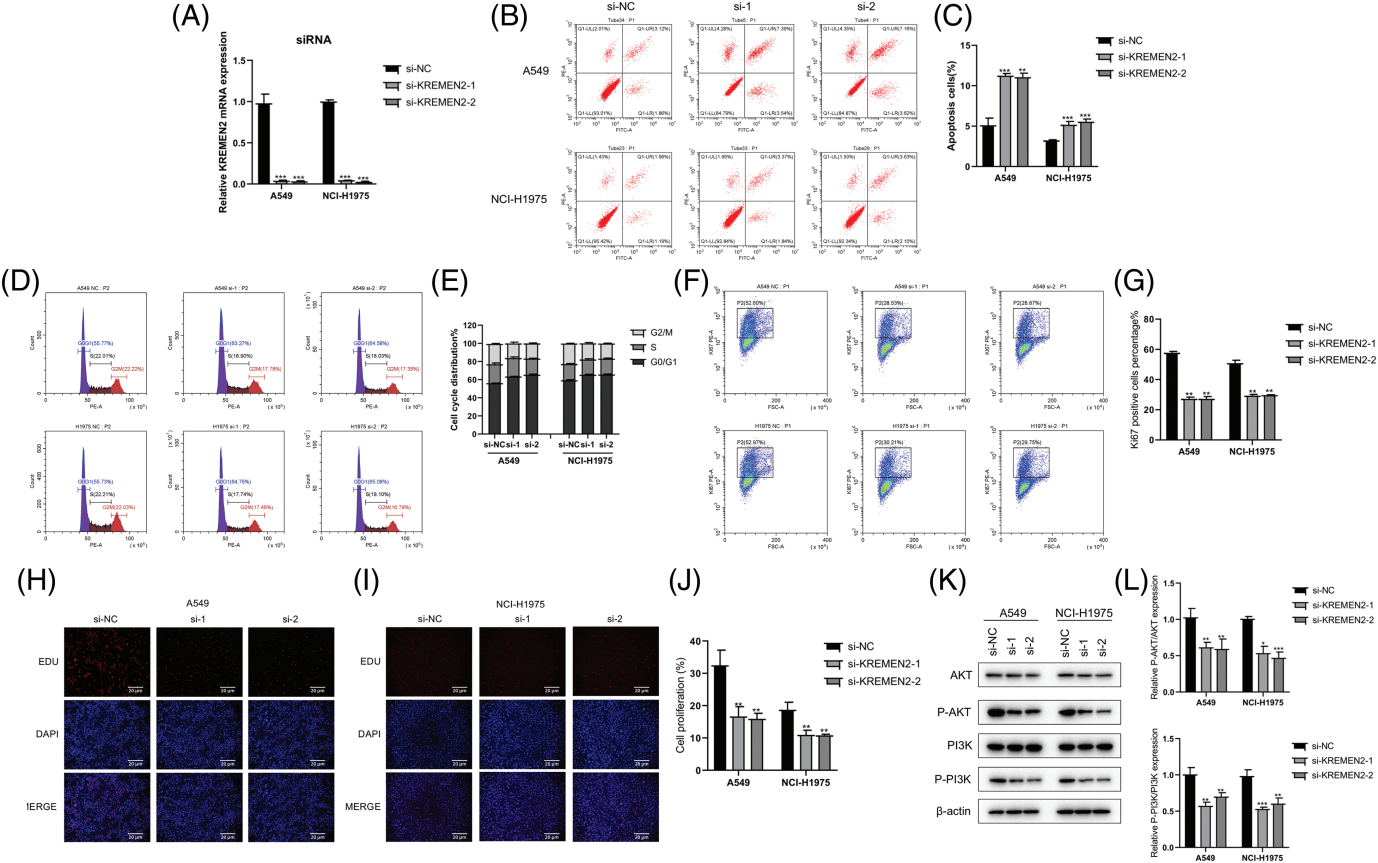
Examination of si-1 and si-2’s impact on apoptosis, cell cycle, transfection efficiency, and protein expression in A549 and NCI-H1975 cells. (A–C). Flow cytometry analysis of apoptosis in A549 and NCI-H1975 cells treated with si-1 or si-2. (D and E). Flow cytometry analysis of the cell cycle in A549 and NCI-H1975 cells treated with si-1 or si-2. (F and G). Transfection efficiency of si-1 and si-2 in A549 and NCI-H1975 cells. The GFP-positive cell rate significantly decreased in si-1 and si-2 groups compared to the si-NC group. (H–J). Hoechst 33342 staining of A549 and NCI-H1975 cells treated with si-1 or si-2. (K and L). Western blot analysis of the expression levels of phosphorylated AKT and PI3K proteins in A549 and NCI-H1975 cells treated with si-1 or si-2. *p < 0.05, **p < 0.01, ***p < 0.001.

In an endeavor to discern if the inhibition of LUAD lung cancer cell growth in A549 and NCI-H1975 by si-1 and si-2 is associated with an enhancement of apoptosis pathway activation, we examined the effect of si-1 and si-2 on apoptosis in A549 and NCI-H1975 cells using flow cytometry. The results demonstrated that si-1 and si-2 induced 10.93% and 10.78% apoptosis in A549 cells (*p* < 0.05), respectively; furthermore, we observed that si-1 and si-2 triggered 5.21% and 5.73% apoptosis in NCI-H1975 cells (*p* < 0.05), respectively, which was statistically significant. Additionally, compared to the si-NC group, the mRNA expression of si-1 and si-2 was markedly downregulated in A549 and NCI-H1975 cells (*p* < 0.05) (Figs. 10A–10C).

### Cell cycle detection after siRNA LUAD

In the cell cycle experiment, colorectal cancer cells A549 and NCI-H1975 transfected with si-1 or si-2 were treated with cell cycle detection kit, and the changes in cell cycle were detected using flow cytometry. Compared with the control group (G0/G1 phase 55.77%, S phase 22.01%, G2/M phase 22.22%), in A549 cells transfected with si-1 and si-2, the proportion of G0/G1 phase significantly increased (63.27%, 64.58%, respectively); the proportion of S phase significantly decreased (18.9%, 18.03%, respectively); the proportion of G2/M phase also decreased (17.78%, 17.35%, respectively). Similarly, in NCI-H1975 cells, we found that after transfection with si-1 and si-2, compared with the control group (G0/G1 phase 55.73%, S phase 22.21%, G2/M phase 22.03%), the proportion of G0/G1 phase increased significantly (64.75%, 65.08%, respectively); the S phase proportion decreased (17.74%, 18.1%, respectively); and the G2/M phase proportion also decreased significantly (17.49%, 16.79%, respectively) (Figs. 10F and 10G). These results suggest that inhibiting KREMEN expression causes cell cycle arrest at G0/G1 in colorectal cancer cells.

### siRNA transfection efficiency

The percentage of GFP-positive cells was statistically significant between the control group and si-1 and si-2 groups (*p* < 0.01) (Figs. 10F and 10G). In A549 cells, compared with si-NC (52.6%), the GFP-positive cell rate in both si-1 and si-2 groups decreased (28.53%, 28.87%, *p* < 0.01, respectively); and in NCI-H1975 cells, compared with si-NC (52.97%), the GFP-positive cell rate in both si-1 and si-2 groups decreased (30.21%, 29.75%, *p* < 0.01, respectively).

### Hoechst 33342 staining of living cells

Apoptotic cells exhibited strong blue fluorescence after Hoechst 33342 staining, and the level of cell apoptosis can be qualitatively determined from the number of positive cells. Staining results in A549 and NCI-H1975 cells showed that compared to the number of positive cells in the control group, the number of positive cells decreased after intervention with si-1 and si-2 (Figs. 10H–10J).

### Western blot detection of phosphorylated protein expression of AKT and PI3K

In A549 cells, we found that the expression of AKT and PI3K after phosphorylation significantly decreased with si-1 and si-2, and the difference was statistically significant compared with the control group (*p* < 0.05). In NCI-H1975 cells, we observed that the levels of p-AKT and p-PI3K proteins decreased after intervention with si-1 and si-2, and the difference was statistically significant compared with si-NC (*p* < 0.05) (Figs. 10K and 10L).

## Discussion

In this investigation, we harnessed the capabilities of Machine Learning (ML) to delve into the profound intricacies of mRNA and mutation signatures associated with drug response in lung adenocarcinoma (LUAD). The significance of addressing drug resistance in LUAD, given its predominant occurrence and the lethal nature among cancers, cannot be overstated. Our findings have shed light on pivotal molecular features that determine the responsiveness of certain drugs, a crucial step towards personalized medicine.

Our usage of the GDSC database, an acclaimed platform in oncogenomics, endowed us with rich datasets, encompassing mRNA expressions, genomic mutations, and drug sensitivities. In comparison to previous studies which typically focused on singular metrics (often just gene expressions or mutations), our holistic approach encompassed both mRNA and mutation profiles, offering a more comprehensive view of the underlying mechanisms.

In the realm of machine learning, the debate over the optimal predictive model is ceaseless. The versatility of Random Forest (RF), the multi-layered sophistication of Artificial Neural Networks (ANN), and the high-dimensional prowess of Support Vector Machines (SVM) have each found their acclaim in distinct applications [31–33]. Our study uniquely positioned them in direct competition under the same conditions. Our discovery that SVM demonstrated exemplary performance in predicting the response for Afuresertib is consistent with previous research suggesting that SVM tends to outperform in cases with high dimensionality and when the relationship between features and response is complex. However, it is noteworthy that ANN, with its deep learning capacities, excelled in predicting Gefitinib’s response, underscoring that no singular model universally dominates in all circumstances.

Comparing our results with similar endeavors, our identification of 1,564 gene features and 45 mutational features for 46 drugs stands out in its granularity. Studies such as those by Smith et al. and Kaur et al. primarily revolved around gene expressions, with a mere focus on a handful of genes, potentially bypassing the intricate interplay of myriad genes and mutations [34,35]. Our approach, in contrast, paints a more holistic picture, setting the foundation for further nuanced research.

While our findings associated with Afuresertib and Gefitinib are promising, the nuances of machine learning predictions must be understood. Our chosen molecular features, like CIT, GAS2L3, and ATP2B4-mut, while instrumental in predictions, are pieces of a complex puzzle. Earlier studies have posited various genes and pathways linked to drug responsiveness, but the interpretation varies based on patient cohorts, data preprocessing, and methodologies adopted [36]. The overlaps and deviations between our results and prior findings should be scrutinized further, potentially unveiling novel insights or corroborating existing hypotheses.

There are limitations to our study that should be acknowledged. Despite the robustness of ML models, they are, in essence, correlative. Our predictions, though highly accurate, might not capture causative relationships. Moreover, the inherent heterogeneity in tumor cells, as stated in our background, means that a ‘one-size-fits-all’ approach might not always be feasible.

In conclusion, our study is a significant stride towards harnessing ML in predicting drug responsiveness in LUAD, laying emphasis on the intricate interplay of mRNA and mutations. By juxtaposing our findings with those from previous research, it becomes evident that the road to conquering drug resistance in lung cancer is convoluted yet achievable, necessitating multifaceted approaches like ours.

## Data Availability

The datasets used during the current study are available from the corresponding author on reasonable request.
